# A Systematic Review of Deep Learning Methodologies Used in the Drug Discovery Process with Emphasis on In Vivo Validation

**DOI:** 10.3390/ijms24076573

**Published:** 2023-03-31

**Authors:** Nikoletta-Maria Koutroumpa, Konstantinos D. Papavasileiou, Anastasios G. Papadiamantis, Georgia Melagraki, Antreas Afantitis

**Affiliations:** 1 Department of ChemoInformatics, NovaMechanics Ltd., Nicosia 1070, Cyprus; 2 School of Chemical Engineering, National Technical University of Athens, 157 80 Athens, Greece; 3 Division of Data Driven Innovation, Entelos Institute, Larnaca 6059, Cyprus; 4 Department of ChemoInformatics, NovaMechanics MIKE., 185 45 Piraeus, Greece; 5 Division of Physical Sciences & Applications, Hellenic Military Academy, 166 73 Vari, Greece

**Keywords:** drug discovery, drug design, artificial intelligence, machine learning, deep learning, biological evaluation, animal model, in vivo

## Abstract

The discovery and development of new drugs are extremely long and costly processes. Recent progress in artificial intelligence has made a positive impact on the drug development pipeline. Numerous challenges have been addressed with the growing exploitation of drug-related data and the advancement of deep learning technology. Several model frameworks have been proposed to enhance the performance of deep learning algorithms in molecular design. However, only a few have had an immediate impact on drug development since computational results may not be confirmed experimentally. This systematic review aims to summarize the different deep learning architectures used in the drug discovery process and are validated with further in vivo experiments. For each presented study, the proposed molecule or peptide that has been generated or identified by the deep learning model has been biologically evaluated in animal models. These state-of-the-art studies highlight that even if artificial intelligence in drug discovery is still in its infancy, it has great potential to accelerate the drug discovery cycle, reduce the required costs, and contribute to the integration of the 3R (Replacement, Reduction, Refinement) principles. Out of all the reviewed scientific articles, seven algorithms were identified: recurrent neural networks, specifically, long short-term memory (LSTM-RNNs), Autoencoders (AEs) and their Wasserstein Autoencoders (WAEs) and Variational Autoencoders (VAEs) variants; Convolutional Neural Networks (CNNs); Direct Message Passing Neural Networks (D-MPNNs); and Multitask Deep Neural Networks (MTDNNs). LSTM-RNNs were the most used architectures with molecules or peptide sequences as inputs.

## 1. Introduction

The key aims to curing diseases using de novo drug development involves the molecular design of new chemical entities with desired properties or the identification of known molecules that can modulate the effect of a disease. The generalized steps in the drug discovery pipeline include target discovery, lead compound discovery and synthesis pathways, and lead optimization [1]. This process can take up to five years, and 5000–10,000 candidate compounds are tested to achieve a single approved drug. On average, it takes 10–15 years with a total cost of $2–3 billion for a new drug to reach the market [2,3]. Once a target has been identified, the pharmaceutical industry and academic centers follow several workflows to identify molecules that possess the characteristics that render them acceptable as drugs [4]. However, the chemical space is vast (i.e., 10^23^–10^60^), and the exploration of a molecule balancing multiple properties, as well as safety and potency against a specific target, is challenging [5].

Computer-aided drug design (CADD) methods have become a powerful tool in the process of drug discovery and development [6]. When the structure of the target is available, structure-based drug design methods can be implemented. Biological target structures can be defined with techniques such as X-ray, NMR, and electron microscopy [7]. When the three-dimensional structure of the target is unknown, a ligand-based strategy such as quantitative structure–activity relationships (QSAR) and pharmacophore modeling [8] can be followed. The increasing amount of available chemical and biological data combined with the evolution of automation technology has generated great efforts to design and apply novel machine learning methodologies in the drug discovery pipeline [1]. All stages of drug discovery have benefited from the utilization of machine learning algorithms and software to identify novel targets, improve small-molecule compound design, etc. [1,2,3,4,5,6,7,8].

Deep learning is a subdivision of machine learning that allows computational models to learn representations of data with multiple levels of abstraction [9]. Big data combined with the more powerful computer hardware that allows faster parallel data processing, such as graphical processing units (GPUs), allows deep learning to be successfully applied in a range of applications. These include, but are not limited to, speech recognition, computer vision, and natural language processing (NLP). The basis of deep architectures is Artificial Neural Network (ANNs) systems with many layers of nonlinear processing units for learning data representations [10]. Deep learning uses a higher number of hidden layers, whereas traditional Neural Networks (NNs) use one or two hidden layers [11]. Each nonlinear module transforms the previous representation into a higher-level representation. With the composition of these architectures, very complex features can be learned [12]. The main deep learning architectures can be classified into one of the three categories: Convolutional Neural Networks (CNNs), Recurrent Neural Networks (RNNs), and Pretrained Unsupervised Networks (PUNs) [12]. CNNs differ from traditional NNs as they perform convolution in at least one of their layers. RNNs allow the connection among neurons in the same hidden layer to form a directed cycle. RNNs can take sequential data, so they are used for time-dependent tasks and language modeling. PUNs are deep learning models that use unsupervised learning to train the hidden layers and achieve better fitting of the data. The two most used PUNs architectures are Generative Adversarial Networks (GANs) and Autoencoders (AEs) [12].

**Figure 1:**
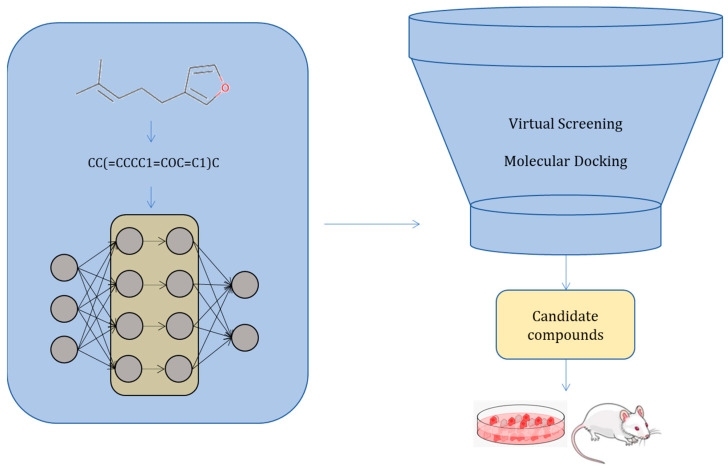
The workflow followed by most studies presented in this review. It contains molecules, molecular encoding, a deep architecture model, virtual screening, and/or molecular docking to reduce the number of candidate compounds to a final set of compounds. These are synthesized and tested for their activity in vitro and in vivo.

Recently, deep learning methods have gained increasing popularity in the fields of cheminformatics, drug discovery, and drug design. By utilizing nonlinear models in the hidden layers of the deep NNs, complex patterns can be extracted, providing a better understanding of the very complex contexts of biological space [13]. Deep learning can be employed for the prediction of drug–target interactions (DTIs), de novo molecular design, synthesis prediction, etc. [14,15,16]. While several studies have reviewed the contribution of deep learning in drug development, in vivo evaluation of the published algorithms is limited. The aim of this systematic review is to enumerate and highlight the deep learning methodologies that have been applied in the drug discovery or drug design process. Our focus is on the models that have identified or generated molecules that have been validated in animal models. Figure 1 depicts the steps of the general workflow followed by the scientific studies covered in this review.

## 2. Materials and Methods

### 2.1. Protocol and Registration

This systematic review was registered on PROSPERO, the international prospective register of systematic reviews, of the National Institute for Health Research. The review question was stipulated as: “Which Deep Learning methodologies have been used for drug design or drug discovery and have been validated with in vivo studies?” The protocol for the systematic review can be found at [17]. The Systematic Reviews and Meta-Analyses (PRISMA) checklist for systematic reviews was applied [18].

### 2.2. Eligibility Criteria

We considered published studies which utilized a deep learning methodology to drug discovery and drug design, and the resulting molecules of the study have been validated in vivo. We considered studies that investigated small molecules and peptides as potential therapeutic candidates for a specific disease. All publications were written in English and published between January 2018 and April 2022. Further details on the characteristics of individual studies are covered in Section 3. Studies that do not include the in vivo evaluation of the selected compounds by the deep learning method were excluded. Studies that did not contain information about the deep learning method developed were also excluded.

### 2.3. Study Information Sources and Search Terms

The sources for performing the literature review were Scopus [19], PubMed [20], SciFinder [21], and Google Scholar [22]. These databases were selected because they contain an abundance of publications and peer-reviewed papers. The search on these databases was completed in April 2022. The terms that were used to search abstracts, titles, and keywords of papers were:

1. (“drug discovery” OR “drug design” OR “de novo” OR “ligand-based” OR “structure-based” OR “virtual screening” OR “protein-ligand interaction *” OR “protein-protein interface”) AND
2. (“deep learning” OR “neural network” OR autoencoder * OR “generative adversarial network” OR “deep reinforcement learning” OR “graph attention”) AND
3. (“in vivo” OR “animal” OR “mouse” OR “murine” OR “rat”)

A correct balance between sensitivity and specificity of the research was identified, in order to maximize high quality data retrieval [23]. A sensitive search provides the researchers with the opportunity to lower the risk of relevant data loss, however, more irrelevant literature is retrieved as well, increasing the time for filtering and screening [23]. On the other hand, specificity decreases the retrieval of irrelevant results and there is a substantial amount of time saving for filtering and screening the results. The drawback is that the more specific the search becomes, the higher the risk of missing relevant literature [23,24]. In the case of this systematic review, an example of a specificity search is the operator is: de novo AND autoencoder. The respective example of a sensitive search is: drug discovery AND neural network AND in vivo. As indicated in Section 2.4, from a total of 283 papers, only 12 were selected for this systematic review. The low percentage of papers that meet the eligibility criteria is attributed to the inclusion of the search term “in vivo”, which dramatically increased the produced results without necessarily increasing the number of eligible papers. Several papers contained the search term “in vivo” without containing an experimental evaluation of the model. On the other hand, removing this specific search term could reduce the number of identified papers with a risk of losing articles of interest. An article could possibly present in vivo studies to verify the in silico results and not describe the selected animal model in the abstract.

### 2.4. Study Selection

**Figure 2:**
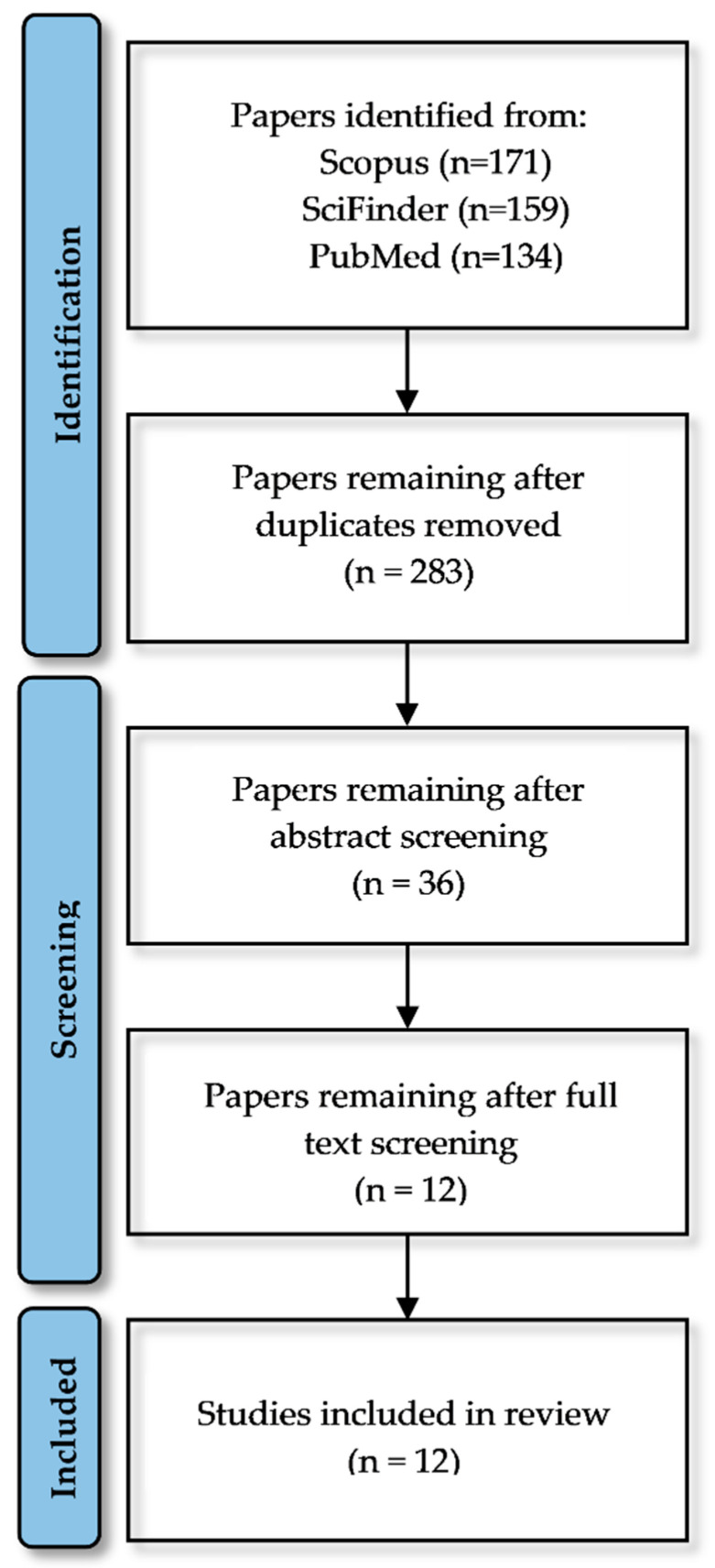
A summary of the papers considered in each stage of the review process. Studies combining early-stage drug discovery and preclinical studies are very limited, resulting in 12 studies to be included in the review.

The titles and abstracts of papers obtained using the search terms presented in Section 2.3 were collected. Publications that did not meet the eligibility criteria were removed. The remaining articles were carefully studied and examined. Those satisfying the inclusion criteria were characterized and included in the present review. A total of 464 papers were initially identified. Following the removal of duplicates, 283 papers remained. All abstracts were screened, resulting in 36 papers that were retained for full text screening. A list of selected papers for full text screening can be found in the Supplementary Material. Although a lot of studies present interesting results on the application of deep learning models in drug design, “real world” application examples of published algorithms are still relatively rare. Only 12 out of 36 papers present a deep learning model whose results are validated in vivo. Most of the retrieved studies presented the possibilities of deep learning in drug discovery, highlighting the importance of further in vitro or/and in vivo evaluation [25]. Other studies took their research one step further and confirmed the in silico results with in vitro experiments [26]. Studies that continued the evaluation of the in silico results with in vivo experiments were scarce. Deep learning models can accelerate, for example, the hit identification and lead optimization steps, which are present in the early drug discovery phase. The in vitro and in vivo studies are integral in the preclinical phase of the drug development pipeline. The existence of both early-stage research and preclinical studies is not very usual and research is probably conducted by scientists from several fields. As a result, the papers that met the criteria of this review—validating the result of a deep learning algorithm with in vivo experiments—are very limited. However, even the existence of those few published papers is essential for the direct evaluation of the contribution of deep learning methods in drug discovery and development. Papers considered in each stage of the review process are shown in Figure 2.

### 2.5. Outcomes

This review includes the deep learning architectures developed in each study, the molecular representation, the selected animal model for the validation of the identified compounds, the drug/compounds reported by each study as the most effective in an animal model, and the pipeline followed in each study.

## 3. Results

We first present the fundamentals of deep learning algorithms, and we review the latest developments in the application of various models in drug discovery. These include not only in silico applications, but also established cases with experimental verification results.

### 3.1. Applied Deep Learning Models Overview

The deep learning models presented here are divided into four categories, including the models based on AEs, GANs, RNNs, and CNNs. The basic principles and recent developments of these models are described together with highlights of their use in drug discovery.

#### 3.1.1. Autoencoders

**Figure 3:**
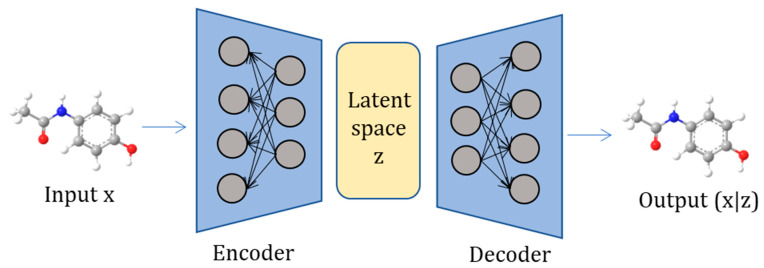
An autoencoder consists of an encoder functionality, which translates an input into a latent space, and a decoder, which translates the internal latent space back to the original input space. The goal of the autoencoder is to compute a reconstruction x’ with minimal error compared to the original input x.

AEs are deep learning structures for unsupervised learning that consist of an encoder and a decoder. They are a type of feed-forward neural network with an extra bias for calculating the error of reconstructing the original input [12]. They use unsupervised learning for dimensionality reduction, compressing the input in the hidden layer, and generating an output that is close to the original input as much as possible (Figure 3). One variant of AEs is the Adversarial Autoencoder (AAE). AAE is a probabilistic autoencoder that uses Generative Adversarial Networks (GANs) to perform variational inference by matching the aggregated posterior distribution of the latent representation of the autoencoder to an arbitrary prior distribution [18]. Adversarial training is used for discriminatively predicting whether samples originate either from hidden code or a user-specified distribution [12]. AAE can be used for semi-supervised classification, unsupervised clustering, dimensionality reduction, etc. [12,27]. A Variational Autoencoder (VAE) assumes that the data are sampled from an arbitrary statistical distribution [28]. It is trained in an unsupervised manner with an encoder that provides a low-dimensional latent representation of the data vector, and a decoder which attempts to reconstruct the input vector. The encoder transforms its input into the parameters of a multidimensional statistical distribution, and sampling occurs where a point is drawn from the encoded distribution and fed into the decoder [28]. It can be seen as a probabilistic version of AE that can generate new data and transform existing data within an encoding–modification–decoding scheme [29]. A VAE which directly encodes from and decodes to discrete data represented as a parse tree from a context-free grammar is called Grammar Variational Autoencoder (GVAE). This architecture ensures that the generated outputs of discrete data are syntactically valid [30].

AEs have been widely used in de novo drug design [31]. The encoder converts the discrete representation of a molecule into a multidimensional continuous representation, and the decoder converts these continuous vectors back to discrete molecular representations. This model allows the exploration of the chemical space through the development of optimized chemical structures. Schultz et al. [32] developed a VAE-based software that generated novel antagonists of the NMDA receptor. Data obtained in silico and experimentally were combined to train and refine the model, improving its predictive accuracy. A conditional VAE was employed to develop a new molecular design strategy that generated molecules with the desired target properties [33]. The AAE is a method that can show good performance in the generation of new compounds while compressing the data to the latent space. An AAE was developed for the identification and generation of new compounds in oncology [34]. The same group compared the VAE and AAE as a molecular generator model in terms of the reconstruction error and variability of the output molecular fingerprints and published an improved model named druGAN [35]. Compared with the VAE model, the AAE model showed better capacity and efficiency in generated new molecules with specific anticancer properties.

#### 3.1.2. Generative Adversarial Networks

**Figure 4:**
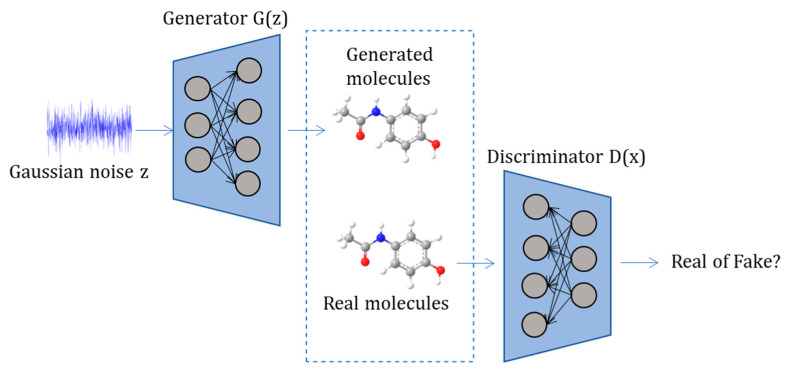
Generative Adversarial Network (GAN): Two independent competing networks are trained simultaneously: the Generator (G), which takes an input z from probability distribution p(z) and generates data G(z); and the Discriminator (D), which receives as input the training data or the output from the generator G(z) and tries to predict whether the input is real or generated.

In GANs, two neural networks are trained simultaneously: the generator and the discriminator (Figure 4). The objective of the generator is to create an output that is so similar to the real one that it makes it difficult for the discriminator to differentiate between real and fake data [10]. GANs have gained attention in applications such as image reconstruction, segmentation, detection, and classification [12,36]. There are various GAN architecture applications used in drug discovery [37]. Sanchez-Lengeling et al. [38] invented the Objective-Reinforced Generative Adversarial Network for Inverse-design Chemistry (ORGANIC)—a framework of previous published Objective-Reinforced Generative Adversarial Networks (ORGAN) architecture [39]. With the exception of solubility, ORGAN performed well in comparison to naïve Reinforcement Learning (RL) in terms of drug likeliness and synthesizability [39]. The main shortcoming of ORGANIC was the large number of invalid molecules and the numerous repetitions in the valid molecules. Another architecture called reinforced adversarial neural computer (RANC) was developed for de novo drug design combining GANs and RL. RANC used a differential neural computer—a type of RNN with external memory—as a generator. The existence of an explicit memory bank mitigated common problems found in adversarial settings [40].

#### 3.1.3. Recurrent Neural Networks

**Figure 5:**
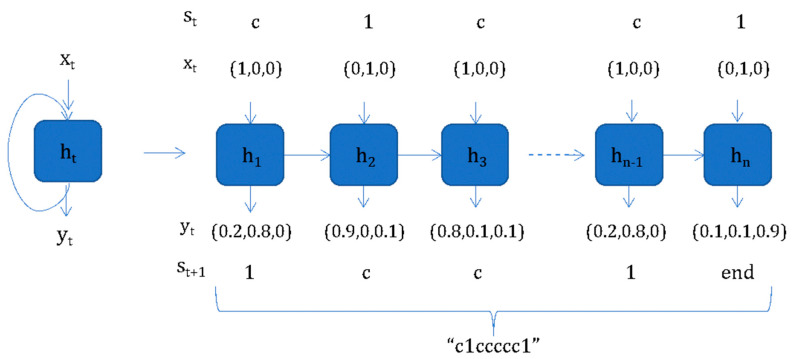
Architecture of recurrent neural networks. The inputs are represented by x_t_. For the standard RNN, the hidden state at time step t is represented as s_t_.; is the “memory” of the network, and for time step t, s_t_ is calculated based on the previous hidden state and the input at the current step: s_t_ = f(Ux_t_ + Ws_(t − 1)_). The function f is usually a nonlinearity, such as tanh or Rectified Linear Unit (ReLU).

RNNs, like feed-forward networks, may not have cycles among conventional edges, but edges that connect adjacent time steps (Figure 5). These are called recurrent edges and introduce a notion of time to the model. RNNs can pass information across sequential steps and process data one element at a time. Thus, the input features can be nondependent sequences of elements [41]. An input is consecutively processed and a connection carrying the output from the previous step into the current step is introduced. As the number of steps increases, RNNs face the problem of vanishing or exploding gradients during backpropagation, thus impairing the training problem. The effect of the input on the hidden layer may decay or blow up, causing the so-called vanishing gradient problem. Many attempts have been made to reduce this problem, with long short-term memory (LSTM) and gated recurrent units (GRU) being the most favored approaches [42,43].

Several examples of the employment of RNNs in de novo drug design have been reported in the literature [44,45,46]. In the work of Olivecrona et al. [44], one of the first attempts to produce a generative model for molecular de novo design is described. A policy-based RL approach was proposed to fine-tune RNNs for generating molecules with given desirable properties. Training of an RNN was performed through maximum likelihood estimations of the next token in a target sequence of given tokens from the previous steps. Once the RNN was trained, it was used to generate new sequences. One year later, Popova et al. [45] proposed a stacked LSTM-RNN model which implemented RL to generate new chemical structures with desired physical and/or biological properties. Transfer learning (TL) approaches were also used to fine-tune the predictions of RNNs for specific molecular targets [47]. Gupta et al. [47] trained an LSTM-RNN model to generate libraries of valid SMILES strings. The model was fine-tuned with TL to generate molecules that were structurally similar to drugs with known bioactivities against a particular biological target. Similar to Popova et al. [45], Gupta et al. combined RNN with another technique to reduce the error and unwanted bias. A scaffold-based deep generative model was proposed by Arús-Pous et al. without the implementation of RL or TL [48]. An LSTM-RNN generated scaffold–decorations tuples and another LSTM-RNN decorated the scaffolds. The trained models became synthetic-chemistry-aware and generated molecules that had synthetically feasible decorations without the need to combine it with other techniques [48].

#### 3.1.4. Convolutional Neural Networks

**Figure 6:**
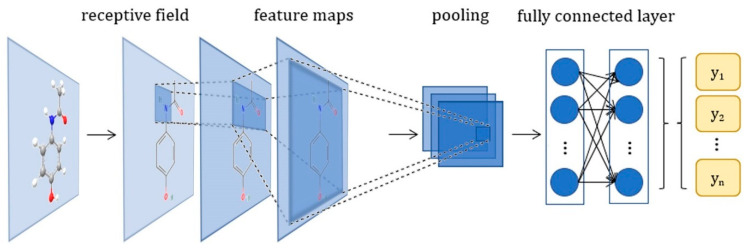
Schematic diagram of a CNN. A convolutional layer followed by a pooling layer forms a convolutional module. Each module learns to identify features while preserving spatial relationships. A fully connected layer is followed, which utilizes the output from the convolution process and predicts the class in a classification problem, based on the features extracted in previous stages.

CNNs are a specialized type of NNs that perform convolution in at least one layer [12]. The first few stages of the CNN have two types of layers: the convolutional layers and the pooling layers (Figure 6). The convolutional layers generate new images called feature maps, and each unit is connected to local patches in the feature map of the previous layer through weights [12]. The created feature maps are processed through a nonlinearity such as the ReLU activation function. The role of the pooling layer is to reduce the size of the input image, as it merges semantically similar features into one. Typically, a pooling unit computes the maximum of a local patch into one feature map. Different convolution, nonlinearity, and pooling stages are stacked, followed by fully connected layers. CNNs have a large range of applications in image classification, video recognition, and image analysis [9,49]. CNNs have been used in drug design to improve the performances of the ligand-based virtual screening process [50] or the prediction of DTIs [51]. An efficient variant of CNNs on graphs is a graph convolutional network (GCN). GCNs stack layers of learned first-order spectral filters followed by a nonlinear activation function to learn graph representations [52]. GCN models are a type of NN that can leverage the graph structure and combine node information from the neighborhoods in a convolutional manner. Ryu et al. [53] proposed an attention- and gate-augmented GCN for the prediction of molecular properties. For the same purpose, an edge-attention-based multirelational GCN was developed [54].

### 3.2. Generating Compounds and Searching Chemical Libraries

The goal of drug discovery is to discover new chemical structures with desired pharmacological properties. De novo molecular design aims to leverage computational methods to automate the molecular generation process and reduce the time of searching in a virtually infinite chemical space [55]. Most existing studies of generative models use the Simplified Molecular Input Line Entry System (SMILES), which is a line notation encoding topological and structural properties of molecules [56], or molecular graphs as molecular representation.

One notable study that includes the biological evaluation of the in silico results was published by Zhavoronkov et al. [57], who introduced a generative tensorial RL pipeline named GENTRL. The model employed the variational inference, tensor decomposition, and RL, combined with three different self-organizing maps (SOMs), which were used as reward functions. GENTRL successfully discovered potent inhibitors of Discoidin domain receptor1 (DDR1). Within 23 days, 30,000 unique and valid structures were obtained using the generative model, and six compounds were selected for further examination. By day 46, these molecules had been synthesized and tested for their in vitro inhibitory activity. One compound was tested in mice and showed favorable pharmacokinetics, demonstrating the potential of the method for effective molecular design [57]. Compounds with a potent DDR1 inhibition profile were also designed by Tan et al. [58]. The authors identified a series of FGFR inhibitors, including compound DC-1, which was selected as a starting point for developing DDR1 inhibitors. A scaffold-based molecular design method was developed, consisting of the matched molecular pairs algorithm proposed by Arús-Pous et al. [48] and an AE as generative model. The most potent compounds were selected based on the kinase selectivity and molecular docking scores [58]. To evaluate the quality of generated molecules, the synthetic accessibility score, the water–octanol partition coefficient (clogP), and the molecular weight of generated molecules were compared with the published DDR1 inhibitors. These properties were consistent with DDR1 inhibitors, showing the ability of the model to design molecules with desired properties. Two promising compounds were selected for synthesis and experimental validation, and one showed promising results in the dextran sulfate sodium-induced mouse colitis model [58].

Another successful deep generative model, apart from AE, is the RNN. After training an RNN with a large number of SMILES sequences, the model can generate valid SMILES strings that may not be present in the training dataset. The LSTM models have exhibited significant improvements over the RNN and tend to replace RNNs in drug discovery [47]. Several studies have applied the strategy of TL, training the model with a larger dataset, and then fine-tuning it with a more specific dataset. An LSTM-based generator can be trained with a chemical database and fine-tuned to generate molecules with desired activity against a target. Yang et al. [59] trained an LSTM-based neural network [60] using 200,000 compounds from ChEMBL database. The model was fine-tuned with a smaller dataset of published p300 inhibitors and macrocycle molecules with potential use in several targets to generate novel p300/CREB-binding protein (CBP) lead compounds. A focused library of 672 chemical structures was generated. After filtering, the top compounds were submitted according to their docking score for visual inspection and further systematic optimization. A potential candidate, B026, showed high inhibitory activity against p300/CBP in animal models of human cancer [59]. Similarly, Tan et al. [61] used an LSTM to design antipsychotic drugs. A pretraining was performed to ensure that the LSTM could generate valid molecules, and then the model was fine-tuned to design molecules that target D_1_/D_2_/5-HT_1A_/5-HT_2A_ receptors. Tan et al. combined the generative model with a multitask deep neural network (MTDNN) to predict whether the generated molecules target multiple G-protein coupled receptors (GPCRs) (bioactivities pIC_50_, pEC_50_). Molecules with high predictive activity were used to expand the fine-tuning set at each iteration during the TL process. The validity of the generated compounds was 97% and the novelty was 87%. The deep discriminative model achieved a test model accuracy expressed as an r^2^ of 0.71 and mean absolute error (MAE) of 0.47 for the IC_50_ dataset and an r^2^ of 0.71 and MAE of 0.54 for the EC_50_ dataset. A hit compound was obtained, and analogs of hit compounds were also designed. The activity profiles of 6 analogs were characterized in vitro. Then, the antipsychotic activities of the selected compounds were studied in the phencyclidine-induced locomotor hyperactivity test in ICR mice, showing good potential for subsequent development [61].

Comparison of the deep generative models for de novo molecular design in [57,59], and [61] reveals that the models are pretrained to learn the general SMILES vocabulary and then fine-tuned to generate DDR1 inhibitors, p300-CBP inhibitors, and GPCR inhibitors, respectively, using a smaller set of specific molecules. Generated compounds showed strong inhibitory activity, with an IC_50_ of 10 nM to DDR1, 1.8 nM to p300/9.5 nM to CBP, and 1.6 nM to 5-HT_1A_ for each study. In order to optimize the generated molecules, Zhavoronkov et al. [57] and Tan et al. [61] combined RL into their models. In [57], the reward of the RL was based on a trending SOM that scored compound novelty, a general kinase SOM to distinguish kinase inhibitors from other molecules, and specific kinase SOM to isolate DDR1 inhibitors. In [61], the MTDNN model provides reward signals to generate more attractive molecules. A different approach was followed in [58]: the selective DDR1 inhibitors were generated using a potent scaffold and applying decorations, resulting in the identification of a compound with a potent DDR1 inhibition profile (IC50 of 10.6 nM). This study implemented a global attention mechanism to assign different focus to the information output from the hidden layers of the RNN.

Although many deep learning models use SMILES to represent molecules [59,61], this notation has limitations. For example, a molecule may be represented by multiple different SMILES strings. Moreover, SMILES may be too simple to deliver the topological information of molecular structures. Molecular graphs intuitively express molecules with 2D topological information and are widely adopted for molecular representation for generative models and predictive models [55]. GCN models in drug-related applications construct graph representations of a molecule that include information about the chemical substructures by summing up all the features of all adjacent atoms [13]. GCNs learn their own expert feature representations directly from the data, and they have been shown to be very capable of capturing complex relationships given sufficient data [62]. A model that belongs to this category was published by Yang et al., who proposed an advanced model which adopts a directed message-passing paradigm for property prediction [62]. The direct-message-passing neural network (D-MPNN) matched or outperformed traditional models that use fixed molecular descriptors or other graph neural networks (GNNs). The main difference implemented into their work was that instead of using messages associated with vertices (atoms), the D-MPNN used messages associated with directed edges (bonds). Stokes et al. [63] utilized this D-MPNN in structure-based antibiotics prediction and became the first reported study where it explored with deep learning a large-scale chemical library for the identification of an antibiotic. A drug library of FDA-approved drugs and additional natural products was screened against *E. coli*, resulting in a training dataset of molecules binarized as hit or non-hit. This dataset was used to train a D-MPNN for a binary classification model that predicts the probability of whether a new compound inhibits the growth of *E. coli* or not. The resulting model achieved a receiver operating characteristic curve–area under the curve (ROC-AUC) of 0.896 on the test data. An ensemble of trained models was used in molecules from the Drug Repurposing Hub [64]. After empirically testing, authors proposed halicin as a candidate antibiotic: a preclinical nitrothiazole under investigation as a treatment for diabetes. In vitro studies showed that halicin had a broad-spectrum bactericidal activity and effectively treated various infections in murine models [63]. Additionally, from a set of >107 million molecules from ZINC and WuXi databases, the model identified eight antibacterial compounds that were structurally distant from known antibiotics. This study by Stokes et al. [63] indicates the potential of applying machine learning in antibiotic discovery, enabling the expansion of the antibiotic arsenal and increasing the rate at which new molecular entities are discovered. Following their paradigm, Wang et al. used the same D-MPNN model [62] for the identification of Ca_v_1.3 antagonists as Parkinson’s-disease-relevant drug candidates [65]. They engineered a cell-based drug discovery platform for multiplexed analysis of Ca_v_1 channel blockers, which was used as a pilot test for high-throughput screening (HTS) of plant essential oils. To identify the putative active constituents of the essential oils, in silico virtual screening was performed and validated with the D-MPNN with an ROC-AUC of 0.978. Experimental testing of five candidate compounds confirmed that sclareol showed Ca_v_1.3 antagonistic activity [65].

Deep learning has also been employed for the prediction of drug efficacy and the underlying pathogenic mechanisms. Using the drugs and the corresponding transcriptional profiles as the input, Zhu et al. [66] developed the deep-learning-based efficacy prediction system (DLEPS), which predicts the drug efficacy from changes in transcriptional profiles. DLEPS utilizes chemical libraries and gene signatures for the identification of candidate disease treatment. In this algorithm, SMILES strings were encoded into a latent space through a GVAE, after passing from a CNN and a dense network was used for the prediction of changes in transcriptional profiles. The changes in transcriptional profiles from both the training and test sets were extremely well fitted with an ROC-AUC around 0.90 and 0.74, respectively. The study explored various gene signature inputs, including a dual up-/down-regulated gene set from obesity studies, a dataset for multiple phenotype manifestations in hyperuricemia, and independent disease stage datasets in nonalcoholic steatohepatitis, resulting in the top drug candidates which were further tested experimentally [66].

### 3.3. De Novo Peptide Generation

For a model to be used in the discovery of drug-like molecules, it must first be trained to sort through the many characteristics of molecules and determine which properties should be retained or suppressed. Similarly, deep learning methods can be used in peptide science to perform various tasks, such as peptide identification, property prediction, and de novo peptide generation [67]. Müller et al. [68] presented a generative LSTM-RNN for combinatorial de novo peptide design. The LSTM-RNN was trained on pattern recognition of helical antimicrobial peptides (AMPs) and the trained model was used for sequence generation, generating 91.4% valid unique sequences. Of these sequences, 82% were predicted to be active AMPs compared to 65% of randomly sampled sequences. This model was used by Bolatchiev et al. [69] for combinatorial de novo AMP design and in vivo evaluation of the most promising generated peptides. The authors differentiated the training set from the original publication presenting the generative model [68] and used all AMPs, not only helical peptides. Using an online tool, the generated novel peptides were categorized to define the AMPs with an accuracy of 87%, resulting in a total of 35 selected sequences from 200 generated sequences. Further computer screening of generated sequences resulted in 5 peptides that were active against various microorganisms and were synthesized for further in vitro and in vivo studies [69]. Apart from sequence-based models like RNNs, VAEs have also been used for peptide generation [70]. Similarly to Bolatchiev et al., Das et al. trained a generative model to design AMPs with low toxicity. They utilized a large unlabeled dataset obtained from UniProt to train a VAE and a Wasserstein autoencoder (WAE). To sample peptides with desired properties, authors fitted a Gaussian mixture density estimator and linear property predictors on latent variables of labeled peptide data. Then, they used a rejection sampling scheme to sample desired latent variables and control the generation of sequences. Das et al. showed that the combination of their VAE framework with molecular dynamics simulations and wet-lab experimentation yielded two novel AMPs within 48 days, highlighting the potential of AEs in peptide drug discovery [70]. This study shows that even training the deep generative AE with a large unlabeled dataset, the latent space is informative of peptide properties. As a result, all AMPs generated are unique, valid, and optimized.

By combining a deep generative model with optimization/searching methods such as genetic algorithms, Bayesian optimization, etc., the generation of peptides can be further improved. Schissel et al. combined a generative model, a prediction model, and a genetic algorithm to generate optimized nuclear-targeting miniproteins [71]. An RNN-based generator was used to produce novel cell-penetrating sequences. A CNN predictor was then used to estimate the activity for a given sequence, and a genetic algorithm was used to optimize the sequence. The generated sequences by the LSTM-RNN model were optimized in the predictor–optimizer loop. The predicted miniproteins where characterized as nontoxic and effectively delivered antisense cargo in animal studies [71]. For the inverse design model, multiple combinations of LSTM and nested LSTM layers were combined, achieving an accuracy of 76%.

### 3.4. Interaction Prediction

Interaction prediction plays a vital role in drug discovery. According to polypharmacology, most drugs have multiple effects on both primary and secondary targets. On the other hand, neural networks can simultaneously learn the properties of many types of data. Thus, by combining deep learning with drug-protein(disease)-based networks, the drug selectivity or the protein promiscuity can be evaluated [72]. DTIs identify the interaction sites between drug compounds and protein targets [73]. Furthermore, protein–protein interactions are particularly important in predicting drug development for precisely locating interacting interfaces in pathway-regulatory approaches, as well as drug–drug interactions (DDIs) for identifying potential side effects and discovering novel applications for finding new uses of existing drugs.

Machine learning methods, especially deep learning, are widely applied to DTI predictions. A crucial step in DTI prediction is the feature extraction step of drug–protein networks. AEs are commonly used for feature extraction. In the studies of [74,75], a stacked AE was used to generate low-dimensional, compressed vectors from the original high-dimensional vectors. Zeng et al. [74] proposed a deep learning methodology for new target identification among known drugs. A stacked AE encoded into low-dimensional feature vectors the relational properties, association information, and topological context of each node of a heterogeneous drug–gene–disease network. Topotecan was identified as a direct inhibitor (with an IC50 = 0.43 μΜ) of human retinoic-acid-receptor-related orphan receptor-gamma t (ROR-γt) with therapeutic effects in a multiple sclerosis mouse model. The proposed model, named deepDTnet, achieved high accuracy (ROC-AUC of 0.963). Similarly, Zhao et al. developed a DTI prediction framework [75]. A stacked AE was used to achieve the optimal mapping of the drug space to the protein space and to obtain low-dimensional feature vectors. The resulting feature vectors integrated the attribute characteristics, interaction information, and the network topology of each target. The low-dimensional feature vectors were used to train the model to obtain the optimal mapping space, and a CNN was used to predict DTIs. The experimental results showed that DLDTI achieved promising performance, with ROC-AUC of 0.917. The new DTIs were identified by ranking candidates according to their optimal mapping space proximity. The predicted targets of tetramethylpyrazine were validated on a novel atherosclerosis model [75].

### 3.5. Databases for Drug Discovery

In this section, we provide a summary of databases used for training the selected models presented above. In Table 1, databases used in Section 3.2, regarding models for de novo molecular design and molecular property prediction, and the size of training datasets, are presented. The ZINC database [76,77] contains a curated collection of commercially available chemical compounds prepared for virtual screening. The new version ZINC-15 contains over 120 million purchasable “drug-like” compounds, and all molecules are in biologically relevant, ready-to-dock formats. The ZINC database is used as a pretraining dataset in [57,61]. Zhavoronkov et al. used the ZINC database for the initial training of a VAE. The pretraining dataset is derived by filtering the ZINC database and removing structures containing atoms other than carbon, nitrogen, oxygen, sulfur, fluorine, chlorine, bromide, and hydrogen. In [61], a collection of molecules from ZINC was used to first train the LSTM model to ensure that it can generate rational “drug-like” molecules. The ZINC-15 database was used by Stokes at al. for virtual screening as well. The authors first trained a model with FDA-approved drugs and predicted the antibiotic activity of >170 million molecules from ZINC-15, identifying eight antibacterial compounds that are structurally distant from known antibiotics [63].

Another commonly used database for drug discovery is ChEMBL [78], which comprises bioactive molecules with drug-like properties. The database provides 5.4 million bioactivity measurements for more than 1 million compounds and 5200 protein targets. The ChEMBL database and Integrity database [79], which are a collection of about half a million bioactive compounds, were used by Zhavoronkov et al. for fine-tuning the VAE to generate DDR1 kinase inhibitors. The ChEMBL database was filtered to contain only DDR/FGFR inhibitors and used as a training set by Tan et al. [58]. A scaffold-based library was used by slicing these inhibitors and obtaining a set of 3603 million scaffold–decoration tuples. ChEMBL was used for pretraining and fine-tuning in [59]. For pretraining, molecules that interact with human “single-protein” targets were retained and by fine-tuning with p300 inhibitors, the LSTM-based molecular generator generated potential p300 inhibitors. Other databases used for the training of deep learning models with a lower frequency are presented in Table 1.

**Table 1:** A list of data used for training models for de novo molecular design and molecular property prediction. The dataset size is presented in number of compounds.

Reference	Dataset	Dataset Size
GENTRL [57]	ZINC	904,801
	Integrity, ChEMBL, literature (DDR1 kinase inhibitors)	1370
	Integrity, ChEMBL (Kinase inhibitors)	23,378
	Integrity, ChEMBL (Non-inhibitors)	16,692
	Integrity (Biological active molecules)	17,000
[58]	ChEMBL (DDR/FGFR inhibitors)	902
[59]	ChEMBL	194,560
	ChEMBL (p300 inhibitors)	135
	ChemBridge [80], Asinex[81]	38,176
[61]	ZINC	310,703
	GLASS [82], Reaxys, SciFinder [21]	10,286
DLEPS [66]	L1000 project—Library of Integrated Network-Based Cellular Signatures [83]	17,051
[63]	FDA (growth inhibition of *E. coli*)	2335
	Drug Repurposing Hub [64]	6111
	WuXi, ZINC	>107 million
[65]	Literature search (Calcium channel blockers) MUV [84]	240 400

The databases used in the studies presented in Section 3.3 and Section 3.4 are shown in Table 2. While there are publicly available datasets for protein informatics with labeled activity, their size is limited. For the generation of peptides with antimicrobial activity, which was the goal in studies of Bolatchiev et al. [69] and Das et al. [70], labeled negative data are often more scarce than positive. Bolatchiev et al. trained an LSTM-based generative model using the APD3 database [85]. The generated sequences were further filtered using online available tools to predict AMPs. A different approach was chosen by Das et al., who trained the generative model using unlabeled sequences from the UniProt DB [86].

Labelled data collected from AmPEP [87], DBAASP [88], ToxinPred [89] were used for training a classifier to distinguish sequences with AMP and non-AMP, toxic and non-toxic. Using a larger training dataset, Das et al. improved the generalizability of the generative model and controlled the generation of desired peptides using a smaller, labelled dataset. In [71], the goal was to generate nuclear-targeting abiotic miniproteins, thus, a more specific database containing cell-penetrating peptides—named CPPSite 2.—was used [90].

Section 3.4 presents models focused on DTI prediction. DrugBank [91] is a comprehensive database that contains molecular information about drugs and was used to collect data in both studies [74,75]. DrugBank contains DTIs, DDIs, drug–disease networks (DDNs), etc. Therapeutic Target DB (TTD) [92] contains information about known therapeutic proteins and nucleic acid targets described in the literature. DrugBank, TTD, and PharmGKB [93] were used in [74] for the DTI network. MetaADEBD [94], CTD [95], SIDER [96], and OFFSIDES [97] are databases containing information on drugs and adverse effects, and were used in [74] to design the drug–side effect network.

### 3.6. Drug Representation

String-based representations are the most frequent option for molecular encoding, among which the SMILES strings are the mostly used drug representation. As it is a sequence-based feature, it can be used as a “sentence” to learn the representations. Many deep generative model techniques have been developed specifically for sequence generation. Therefore, when generative models are applied to de novo drug design, SMILES are most used as a molecular representation. An important feature of SMILES is that it is easy to learn and human-readable compared to other methods of molecular representation. Molecules are represented as SMILES strings in studies [58,59,61]. The deep generative models presented in these studies use an LSTM-based network for the design of novel molecules. Tan et al. [61] used canonical SMILES as an input in the generative model and molecular-fingerprint-based descriptors in the discriminative model. A few years later, Tan et al. [58] used randomized SMILES, as it was shown from previous studies that the model trained with randomized SMILES could generate more unique molecules than the model trained with canonical SMILES [98]. Molecules were represented using the SMILES format in other studies as well [57,66]. In [66], the authors tried different ways of encoding SMILES of chemical compounds. They encoded the compounds into latent space as plain text through a VAE, and they also converted them into a grammar tree (GVAE), resulting in the latter being indicated as the best representation. Among the models included in this review, there were studies that focused on de novo peptide design. In these cases, peptide sequences were used as text input to train the models that are learning sequence grammar [69,70,71]. Schissel et al. [71] trained a CNN to predict the activity of sequences, apart from the generative model. For the training of the classification model, one-hot encodings and fingerprints were examined. It was shown that the CNN-fingerprint model was able to extrapolate in the codomain and generate predicted activity values that were greater than any in the training set.

In the study of Stokes et al. [63] and Wand et al. [65], the molecular graph for each molecule was constructed using SMILES strings, following the initial work of Yang et al. [62]. A feature vector was initialized for each atom and bond, based on computable features. The message-passing paradigm followed was based on updating representations of directed bonds rather than atoms. Even though the message-passing paradigm can extract features that depend on local chemistry, it may struggle to extract global features. For that reason, the molecular representation was a concatenation of learned features and fixed molecule-level features. In the cases of interaction prediction, more complex, heterogeneous networks were examined. A deep neural network for graph representation algorithms was employed to learn a low-dimensional vector representation of drugs and targets. The drug–target network was described as a bipartite graph G(D,T,P), where the drug set was denoted as D, the target set as T, and the interaction set as P [74]. In [75], heterogeneous data were integrated, including circular fingerprints to map the structural information of drugs, sequences of drug targets, and graph-embedding-based features for drug and targets.

## 4. Discussion

The selected studies in this systematic review include different applications of deep learning in drug discovery, with their in vivo evaluation results, from de novo molecular design, de novo peptide design, and specifically, AMPs and miniproteins, antibiotic discovery, drug repurposing, and drug efficacy. The available codes and tools of these studies are presented in Table 3.

Deep generative models based on AEs and RNNs are employed for the design of novel molecules and peptides. Most de novo design tasks require generating compounds that meet specific requirements. To optimize the generated compounds, methods such as fine-tuning, TL, and RL have been combined with the core of the generative model. A method that is not examined by the selected studies is the conditional generative model. Conditional molecular design samples new molecules from a conditional generative distribution without any additional optimization process. In the case of [57] and [59], the generated molecules were reduced to most “drug-like” molecules by adding restrictions, such as molecular weight, logP values, no violation of Lipinski’s rule of five, etc. With conditional design, these models could directly produce molecules with desired features. An interesting approach was presented by Das et al. [70], who did not implement an RL approach to design AMPs, since this method for targeted generation requires optimal policy learning. Instead, they trained on the latent space of a deep AE, which represented all known peptide sequences and not only AMPs, an attribute classifier to select the informative space for sampling. This study revealed that the latent space is linearly separable into different functional attributes, and sampling from the selected space can generate optimized peptides. By combining deep generative models with optimization methods such as genetic algorithms, generated samples can be further optimized to acquire improved functions. Schissel et al. [71] studied this notion to generate peptides using a deep generative model with a genetic algorithm. They also examined the representation of amino acids and concluded that topological fingerprints led to models with lower accuracies, but with an enhanced generalizability to peptides with labels outside the range of the training data. Regarding molecular representation for property prediction, D-MPNN [62] which combines fixed and learned features of molecules, was selected both by [63,65]. The hybrid representation of molecules yielded higher performance and generalized better than either convolutional or fingerprint-based models. These studies experimentally evaluated the results of virtual screening using the D-MPNN. In [63] the authors identified an antibiotic that, even if structurally divergent from conventional antibiotics, displays growth-inhibitory properties against a wide spectrum of pathogens. In [65] the authors identified an essential oil that inhibits Ca_v_1.3. Heterogeneous data sources of DTIs, DDNs, PPIs, etc., were fed into AEs for the generation of low-dimensional but informative vectors for both drugs and targets [74,75].

With his methodology, Zeng et al. [74] uncovered known drug targets contributing to drug repurposing. Relationship-based features were collected by training an AE and were used in [75] for DTI prediction. A CNN prediction model that combined deep information was taught using the stacked AE technique.

It is important to emphasize that machine learning is imperfect. Therefore, the success of deep neural network model-guided drug discovery rests heavily on coupling these approaches with appropriate experiments. Before in vitro studies, the results of the deep learning models were filtered based on other methods. Generated molecules were evaluated using SOMs and pharmacophore modeling on the basis of crystal structures in complex with DDR1 [57], kinase selectivity, and molecular docking [58,59]. Generated peptides were screened for AMPs, toxicity, drug efficacy using sequence-level classifiers [70], and online prediction tools [69]. For in vivo studies, the animal model used for the biological evaluation of the compound of interest, and the compound identified by the deep learning algorithm, are presented in Table 4 and Table 5, respectively.

**Figure 7:**
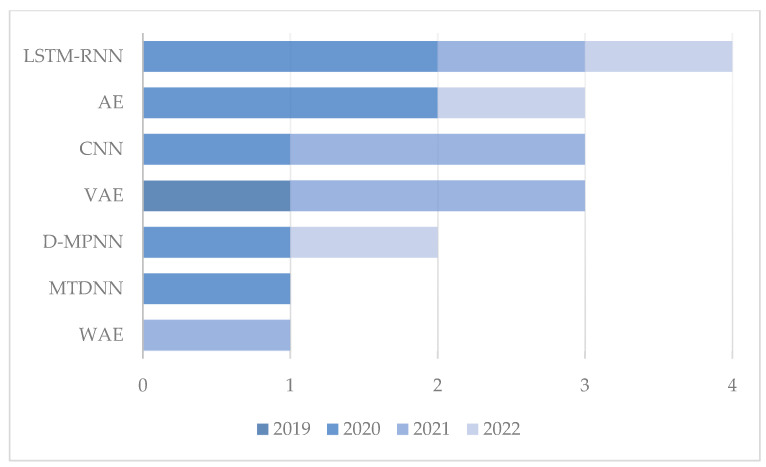
The relative frequencies per year of the deep learning models described in the present review.

Changes in the frequencies of the selected model architectures per year are shown in Figure 7. LSTM-RNN models were the most commonly published models between 2019 and 2022. The four LSTM-RNN models presented include the use of de novo design for antipsychotic drugs [61], p300 and CBP lead compounds [59], nuclear-targeting miniproteins [71], and AMPs design [69]. AEs were widely used in different architectures, including VAEs, GVAEs, and WAEs. Stacked AEs were used to generate low-dimensional vectors from the original high-dimensional vectors [74,75], and AE architectures were used to design DDR1 inhibitors [57,58]. A WAE was used as an alternative to a VAE for AMP design [70], and in the same year, a GVAE was used to encode SMILES into latent space, with this vector then passing through a CNN to estimate the activity of the given sequence [66]. CNNs were used for the activity prediction of the generated sequences [71] and for the prediction of DTIs [75]. A multitask DNN was used for the virtual screening of molecules based on their activity score [61]. D-MPNN was presented in two studies, for predicting the probability of whether a new compound inhibits the growth of a spectrum of pathogens or not [63], and for the identification of Parkinson’s-disease-relevant drug candidates [65].

## 5. Conclusions

Drug discovery based on artificial intelligence has received much attention, since it has had a significant influence on developing novel drugs. Owing to the rapid advancements in computer hardware, coupled with the growth in size and availability of publicly accessible datasets, deep learning has met unprecedented success in the field of CADD [99,100]. Advances in deep learning techniques have been successfully combined with well-established drug design strategies, such as drug repositioning, opening new pathways and prospects in the identification of novel therapeutics using cutting-edge computational methods [101,102]. Particularly in the field of de novo drug design, deep learning applications have gained increasing popularity, since numerous approaches (e.g., RNNs, AEs, GCNs) have been developed to build novel compounds with desired pharmacological and physiochemical properties [103,104].

In the present study, a systematic review of peer-reviewed research articles from 2018 up until April of 2022 is presented. The scientific articles considered were used to extract information regarding trends in deep learning models for drug design that were complemented with in vivo animal studies. The outcomes of this review include the deep learning architectures developed, the molecular representations, the workflow of each study, the animal model for the validation of the selected compounds, and the resulting compounds. The deep learning algorithms that were selected were LSTM-RNNs, AEs, CNNs, MTDNNs, and D-MPNNs. LSTM-RNNs were the more frequently used algorithms.

It is important to note that although several studies have examined the potential role that deep learning models could play in the discovery of new drugs, applications of these models in “real cases” are still uncommon due to the need for additional computational and experimental validation. This review selected breakthrough studies that started from a deep learning model and continued to in vivo studies to provide a validated process. We believe that deep learning will become an essential part in drug discovery in the near future, and as highlighted by the presented studies, and will assist medicinal chemists in generating new ideas and accelerate the cycle of drug discovery.

## Figures and Tables

**Table 2:** A list of data used for training models for de novo peptide design and DTI prediction.

Reference	Dataset	Dataset Size
[69]	APD3	3100 sequences
[71]	CPPSite2.0	1150 19,800 sequence-next character pairs
CLaSS [70]	Uniprot DB AmPEP/DBAASP/ToxinPred	~1.7 million sequences 9000 sequences
deepDTnet [74]	DrugBank/TTD/PharmGKB	5680 DTIs
	15 bioinformatics DBs	16,133 PPIs
	DrugBank	132,768 DDIs
	repoDB, DrugBank, DrugCentral	1208 DDNs
	MetaADEBD, CTD, SIDER, OFFSIDES	263,805 DSENs
DLDTI [75]	DrugBank	904 drugs 613 targets

**Table 3:** Open-source codes and web applications for different tasks of computational drug discovery presented in this systematic review.

Author	Application	Tool
Bolatchiev et al., 2022 [69]	De novo peptide design	github.com/alexarnimueller/LSTM_peptides (accessed on 10 December 2022)
Wang et al., 2022 [65]	Drug property prediction	github.com/chemprop/chemprop (accessed on 10 December 2022)
Das et al., 2021 [70]	De novo peptide design	github.com/IBM/controlled-peptide-generation (accessed on 10 December 2022)
Schissel et al., 2021 [71]	De novo peptide design	github.com/learningmatter-mit/peptimizer (accessed on 10 December 2022)
Zhu et al., 2021 [66]	Drug efficacy prediction	github.com/kekegg/DLEPS www.dleps.tech/dleps/index (accessed on 10 December 2022)
Stokes et al., 2020 [63]	Drug property prediction	github.com/chemprop/chemprop chemprop.csail.mit.edu/ (accessed on 10 December 2022)
Zeng et al., 2020 [74]	DTI	github.com/ChengF-Lab/deepDTnet (accessed on 10 December 2022)
Zhavoronkov et al., 2019 [57]	De novo molecular design	github.com/insilicomedicine/gentrl (accessed on 10 December 2022)

**Table 4:** A selection of the animal models used in each study for biological evaluation.

Author	Animal Model
Tan et al., 2022 [58]	Dextran sulfate sodium-induced inflammatory bowel disease mouse model
Bolatchiev et al., 2022 [69]	Murine experimental model of sepsis
Wang et al., 2022 [65]	Parkinson’s disease mouse model
Das et al., 2021 [70]	BALB/c mice
Schissel et al., 2021 [71]	Mice containing EGFP IVS2-654 gene
Zhu et al., 2021 [66]	Diet-induced obesity mouse model Hyperuricemia mouse model Nonalcoholic steatohepatitis mouse model
Stokes et al., 2020 [63]	Murine wound model of *A. baumannii* and *C. difficile* infections
Tan et al., 2020 [61]	Phencyclidine-induced hyperactivity ICR mouse model
Yang et al., 2020 [59]	Animal model of human cancer (Balb/c mice bearing MV-4-11 tumor cells)
Zhao et al., 2020 [75]	Ldlr−/− hamsters developed severe hyperlipidemia and atherosclerosis lesions
Zeng et al., 2020 [74]	Experimental autoimmune encephalomyelitis mouse model
Zhavoronkov et al., 2019 [57]	C57BL/6 mice

**Table 5:** A selection of reported candidate compounds and biologics in the studies reviewed.

Author	Reported Candidate Compounds and Biologics
Tan et al., 2022 [58]	2-(2-(4-Acetamidophenyl)-4-amino-7-oxo-6,7-dihydro- 2H-pyrazolo[3,4-d]pyridazin-3-yl)-3-methyl-N-(3- (trifluoromethyl)phenyl)benzofuran-6-carboxamide
Bolatchiev et al., 2022 [69]	PEP-36 GIFSKLAGKKIKNLLISGLKNIGKEVGM PEP-137 KWKSFIKKLAKFGFKVIKKFAKKHGSKIAKNQ
Wang et al., 2022 [65]	Sclareol
Das et al., 2021 [70]	YI12 YLRLIRYMAKMI-CONH2 FK13 FPLTWLKWWKWKK-CONH2
Schissel et al., 2021 [71]	Mach3 and Mach4
Zhu et al., 2021 [66]	Chikusetsusaponin IV Perillen Trametinib
Stokes et al., 2020 [63]	c-Jun N-terminal kinase inhibitor SU3327 (halicin)
Tan et al., 2020 [61]	1-(4-(4-(benzo[b]thiophen-4-yl)piperazin-1-yl)butyl)quinazoline-2,4(1H, 3H)-dione
Yang et al., 2020 [59]	(S)-1-(2-((S)-7-Fluoro-3-(trifluoromethyl)-2,3-dihydrobenzo[f ]-[1,4]oxazepin-4(5H)-yl)-2-oxoethyl)-5′-(1-methyl-1H-pyrazol-4-yl)- 2′,3′-dihydrospiro[imidazolidine-4,1′-indene]-2,5-dione (B026)
Zhao et al., 2020 [75]	288 predicted targets of tetramethylpyrazine on atherosclerosis, and 190 proteins involved in the platelet activation process, indicating that tetramethylpyrazine inhibited signaling transduction.
Zeng et al., 2020 [74]	Topotecan

## Supplementary Materials

The following supporting information can be downloaded at: https://www.mdpi.com/article/10.3390/ijms24076573/s1.

## Abbreviations

AAE	Adversarial autoencoder
AEs	Autoencoders
AMP	Antimicrobial peptide
ANNs	Artificial neural networks
CADD	Computer-aided drug design
CBP	CREB-binding protein
CNNs	Convolutional neural networks
D-MPNN	Direct-message-passing neural network
DB	Database
DDIs	Drug–Drug Interactions
DDR1	Discoidin domain receptor1
DDNs	Drug–Disease Networks
DSENs	Drug–Side effect Networks
DTIs	Drug–target interactions
FDA	Food and drug administration
GANs	Generative adversarial networks
GAT	Graph attention network
GCNs	Graph convolutional networks
GNN	Graph neural network
GPCR	G-protein coupled receptor
GPU	Graphical processing units
GRU	Gated recurrent units
GVAE	Grammar variational autoencoder
HTS	High-throughput screening
IC50	Half-maximum inhibitory concentration
LSTM	Long short-term memory
MAE	Mean absolute error
MTDNN	Multitask deep neural network
NLP	Natural language processing
NNs	Neural networks
ORGAN	Objective-Reinforced Generative Adversarial Networks
ORGANIC	Objective-Reinforced Generative Adversarial Network for Inverse-design Chemistry
PPIs	Protein–Protein Interactions
PRISMA	Preferred reporting items for systematic review and meta-analyses
PUNs	Pretrained unsupervised networks
QSAR	Quantitative Structure–Activity Relationships
RANC	Reinforced adversarial neural computer
ReLU	Rectified linear unit
RL	Reinforcement Learning
RNNs	Recurrent neural networks
ROC-AUC	Receiver operating characteristic curve-area under curve
ROR-γt	Retinoic-acid-receptor related orphan receptor-gamma t
SMILES	Simplified molecular input line entry system
SOM	Self-organizing map
TL	Transfer Learning
TTD	Therapeutic Target DB
VAE	Variational autoencoder
WAE	Wasserstein autoencoder

## References

[1] Patel L., Shukla T., Huang X., Ussery D.W., Wang S. (2020). Machine Learning Methods in Drug Discovery. Molecules.

[2] Torjesen I. (2015). Drug Development: The Journey of a Medicine from Lab to Shelf. Pharm. J..

[3] Scannell J.W., Blanckley A., Boldon H., Warrington B. (2012). Diagnosing the decline in pharmaceutical R&D efficiency. Nat. Rev. Drug Discov..

[4] Hughes J.P., Rees S., Kalindjian S.B., Philpott K.L. (2011). Principles of early drug discovery. Br. J. Pharmacol..

[5] Polishchuk P.G., Madzhidov T.I., Varnek A. (2013). Estimation of the size of drug-like chemical space based on GDB-17 data. J. Comput. Mol. Des..

[6] Mouchlis V.D., Melagraki G., Zacharia L.C., Afantitis A. (2020). Computer-Aided Drug Design of β-Secretase, γ-Secretase and Anti-Tau Inhibitors for the Discovery of Novel Alzheimer’s Therapeutics. Int. J. Mol. Sci..

[7] Schneider G., Clark D.E. (2019). Automated De Novo Drug Design: Are We Nearly There Yet?. Angew. Chem. Int. Ed..

[8] Mouchlis V.D., Afantitis A., Serra A., Fratello M., Papadiamantis A.G., Aidinis V., Lynch I., Greco D., Melagraki G. (2021). Advances in de Novo Drug Design: From Conventional to Machine Learning Methods. Int. J. Mol. Sci..

[9] LeCun Y., Bengio Y., Hinton G. (2015). Deep learning. Nature.

[10] Yang X., Wang Y., Byrne R., Schneider G., Yang S. (2019). Concepts of Artificial Intelligence for Computer-Assisted Drug Discovery. Chem. Rev..

[11] Chen H., Engkvist O., Wang Y., Olivecrona M., Blaschke T. (2018). The rise of deep learning in drug discovery. Drug Discov. Today.

[12] Hosseini M.P., Lu S., Kamaraj K., Slowikowski A., Venkatesh H.C., Pedrycz W., Chen S.M. (2020). Deep Learning Architectures. Deep Learning: Concepts and Architectures.

[13] Kim J., Park S., Min D., Kim W. (2021). Comprehensive Survey of Recent Drug Discovery Using Deep Learning. Int. J. Mol. Sci..

[14] Vamathevan J., Clark D., Czodrowski P., Dunham I., Ferran E., Lee G., Li B., Madabhushi A., Shah P., Spitzer M. (2019). Applications of machine learning in drug discovery and development. Nat. Rev. Drug Discov..

[15] Born J., Manica M. (2021). Trends in Deep Learning for Property-driven Drug Design. Curr. Med. Chem..

[16] Kimber T., Chen Y., Volkamer A. (2021). Deep Learning in Virtual Screening: Recent Applications and Developments. Int. J. Mol. Sci..

[17] Koutroumpa N.-M., Afantitis A., Papadiamantis A.G., Melagraki G. A Systematic Review of Deep Learning Methodologies Used in the Drug Discovery Process with Emphasis on the In Vivo Validation. https://www.crd.york.ac.uk/prospero/display_record.php?ID=CRD42022329949.

[18] Page M.J., McKenzie J.E., Bossuyt P.M., Boutron I., Hoffmann T.C., Mulrow C.D., Shamseer L., Tetzlaff J.M., Akl E.A., Brennan S.E. (2021). The PRISMA 2020 Statement: An Updated Guideline for Reporting Systematic Reviews. BMJ.

[19] Scopus. https://www.scopus.com/.

[20] PubMed.gov. National Library of Medicine, National Center for Biotechnology Information. https://pubmed.ncbi.nlm.nih.gov/.

[21] SciFinder. https://scifinder.cas.org.

[22] Google Scholar. https://scholar.google.com/.

[23] University of Toronto Libraries Searching the Literature: A Guide to Comprehensive Searching in the Health Sciences: Precision vs. Sensitivity—Key to Effective Searching. https://guides.library.utoronto.ca/c.php?g=577919&p=4304403.

[24] Relevo R. (2012). Chapter 4 of Methods Guide for Medical Test Reviews. Effective Search Strategies for Systematic Reviews of Medical Tests.

[25] Arshia A.H., Shadravan S., Solhjoo A., Sakhteman A., Sami A. (2021). De novo design of novel protease inhibitor candidates in the treatment of SARS-CoV-2 using deep learning, docking, and molecular dynamic simulations. Comput. Biol. Med..

[26] Khanna V., Li L., Fung J., Ranganathan S., Petrovsky N. (2019). Prediction of novel mouse TLR9 agonists using a random forest approach. BMC Cell Biol..

[27] Makhzani A., Shlens J., Jaitly N., Goodfellow I., Frey B. (2015). Adversarial Autoencoders. arXiv.

[28] Cinelli L.P., Marins M.A., da Silva E.A.B., Netto S.L. (2021). Variational Autoencoder. Variational Methods for Machine Learning with Applications to Deep Networks.

[29] Girin L., Leglaive S., Bie X., Diard J., Hueber T., Alameda-Pineda X. (2021). Dynamical Variational Autoencoders: A Comprehensive Review. Found. Trends Mach. Learn..

[30] Kusner M.J., Paige B., Hernández-Lobato J.M. Grammar Variational Autoencoder. Proceedings of the 34th International Conference on Machine Learning.

[31] Gómez-Bombarelli R., Wei J.N., Duvenaud D.K., Hernandez-Lobato J.M., Sánchez-Lengeling B., Sheberla D., Aguilera-Iparraguirre J., Hirzel T.D., Adams R.P., Aspuru-Guzik A. (2018). Automatic Chemical Design Using a Data-Driven Continuous Representation of Molecules. ACS Central Sci..

[32] Schultz K.J., Colby S.M., Yesiltepe Y., Nuñez J.R., McGrady M.Y., Renslow R.S. (2020). Application and assessment of deep learning for the generation of potential NMDA receptor antagonists. Phys. Chem. Chem. Phys..

[33] Lim J., Ryu S., Kim J.W., Kim W.Y. (2018). Molecular generative model based on conditional variational autoencoder for de novo molecular design. J. Chemin-.

[34] Kadurin A., Aliper A., Kazennov A., Mamoshina P., Vanhaelen Q., Khrabrov K., Zhavoronkov A. (2017). The cornucopia of meaningful leads: Applying deep adversarial autoencoders for new molecule development in oncology. Oncotarget.

[35] Kadurin A., Nikolenko S., Khrabrov K., Aliper A., Zhavoronkov A. (2017). druGAN: An Advanced Generative Adversarial Autoencoder Model for de Novo Generation of New Molecules with Desired Molecular Properties in Silico. Mol. Pharm..

[36] Yi X., Walia E., Babyn P. (2019). Generative adversarial network in medical imaging: A review. Med Image Anal..

[37] Lin E., Lin C.-H., Lane H.-Y. (2020). Relevant Applications of Generative Adversarial Networks in Drug Design and Discovery: Molecular De Novo Design, Dimensionality Reduction, and De Novo Peptide and Protein Design. Molecules.

[38] Sanchez-Lengeling B., Outeiral C., Guimaraes G.L., Aspuru-Guzik A. (2017). Optimizing distributions over molecular space: An objective-reinforced generative adversarial network for inverse-design chemistry (ORGANIC). ChemRxiv.

[39] Guimaraes G.L., Sanchez-Lengeling B., Outeiral C., Farias P.L.C., Aspuru-Guzik A. (2017). Objective-Reinforced Generative Adversarial Networks (ORGAN) for Sequence Generation Models. arXiv.

[40] Putin E., Asadulaev A., Vanhaelen Q., Ivanenkov Y., Aladinskaya A.V., Aliper A., Zhavoronkov A. (2018). Adversarial Threshold Neural Computer for Molecular de Novo Design. Mol. Pharm..

[41] Lipton Z.C., Berkowitz J., Elkan C.A. (2015). Critical Review of Recurrent Neural Networks for Sequence Learning. arXiv.

[42] Hochreiter S., Schmidhuber J. (1997). Long short-term memory. Neural Comput..

[43] Graves A., Graves A. (2012). Long short-term memory. Supervised Sequence Labelling with Recurrent Neural Networks.

[44] Olivecrona M., Blaschke T., Engkvist O., Chen H. (2017). Molecular de-novo design through deep reinforcement learning. J. Chemin.

[45] Popova M., Isayev O., Tropsha A. (2018). Deep reinforcement learning for de novo drug design. Sci. Adv..

[46] Popova M., Shvets M., Oliva J., Isayev O. (2019). MolecularRNN: Generating realistic molecular graphs with optimized properties. arXiv.

[47] Gupta A., Müller A.T., Huisman B.J.H., Fuchs J.A., Schneider P., Schneider G. (2018). Generative Recurrent Networks for De Novo Drug Design. Mol. Inf..

[48] Arús-Pous J., Patronov A., Bjerrum E.J., Tyrchan C., Reymond J.-L., Chen H., Engkvist O. (2020). SMILES-based deep generative scaffold decorator for de-novo drug design. J. Chemin..

[49] Kim P. (2017). Convolutional Neural Network. MATLAB Deep Learning.

[50] Berrhail F., Belhadef H., Haddad M. (2022). Deep Convolutional Neural Network to improve the performances of screening process in LBVS. Expert Syst. Appl..

[51] Huang K., Fu T., Glass L.M., Zitnik M., Xiao C., Sun J. (2020). DeepPurpose: A deep learning library for drug–target interaction prediction. Bioinformatics.

[52] Wu F., Souza A., Zhang T., Fifty C., Yu T., Weinberger K. Simplifying Graph Convolutional Networks. Proceedings of the 36th International Conference on Machine Learning.

[53] Ryu S., Lim J., Hong S.H., Kim W.Y. (2018). Deeply learning molecular structure-property relationships using attention- and gate-augmented graph convolutional network. arXiv.

[54] Shang C., Liu Q., Chen K.S., Sun J., Lu J., Yi J., Bi J. (2018). Edge Attention-based Multi-Relational Graph Convolutional Networks. arXiv.

[55] Sun M., Zhao S., Gilvary C., Elemento O., Zhou J., Wang F. (2019). Graph convolutional networks for computational drug development and discovery. Brief. Bioinform..

[56] Weininger D. (1988). SMILES, a chemical language and information system. Introduction to methodology and encoding rules. J. Chem. Inf. Model..

[57] Zhavoronkov A., Ivanenkov Y.A., Aliper A., Veselov M.S., Aladinskiy V.A., Aladinskaya A.V., Terentiev V.A., Polykovskiy D.A., Kuznetsov M.D., Asadulaev A. (2019). Deep learning enables rapid identification of potent DDR1 kinase inhibitors. Nat. Biotechnol..

[58] Tan X., Li C., Yang R., Zhao S., Li F., Li X., Chen L., Wan X., Liu X., Yang T. (2021). Discovery of Pyrazolo[3,4-*d*]pyridazinone Derivatives as Selective DDR1 Inhibitors via Deep Learning Based Design, Synthesis, and Biological Evaluation. J. Med. Chem..

[59] Yang Y., Zhang R., Li Z., Mei L., Wan S., Ding H., Chen Z., Xing J., Feng H., Han J. (2020). Discovery of Highly Potent, Selective, and Orally Efficacious p300/CBP Histone Acetyltransferases Inhibitors. J. Med. Chem..

[60] Segler M.H.S., Kogej T., Tyrchan C., Waller M.P. (2017). Generating Focused Molecule Libraries for Drug Discovery with Recurrent Neural Networks. ACS Central Sci..

[61] Tan X., Jiang X., He Y., Zhong F., Li X., Xiong Z., Li Z., Liu X., Cui C., Zhao Q. (2020). Automated design and optimization of multitarget schizophrenia drug candidates by deep learning. Eur. J. Med. Chem..

[62] Yang K., Swanson K., Jin W., Coley C., Eiden P., Gao H., Guzman-Perez A., Hopper T., Kelley B., Mathea M. (2019). Analyzing Learned Molecular Representations for Property Prediction. J. Chem. Inf. Model..

[63] Stokes J.M., Yang K., Swanson K., Jin W., Cubillos-Ruiz A., Donghia N.M., Macnair C.R., French S., Carfrae L.A., Bloom-Ackermann Z. (2020). A Deep Learning Approach to Antibiotic Discovery. Cell.

[64] Corsello S., Bittker J.A., Liu Z., Gould J., McCarren P., Hirschman J.E., Johnston S.E., Vrcic A., Wong B., Khan M. (2017). The Drug Repurposing Hub: A next-generation drug library and information resource. Nat. Med..

[65] Wang H., Xie M., Rizzi G., Li X., Tan K., Fussenegger M. (2022). Identification of Sclareol As a Natural Neuroprotective Cav1.3-Antagonist Using Synthetic Parkinson-Mimetic Gene Circuits and Computer-Aided Drug Discovery. Adv. Sci..

[66] Zhu J., Wang J., Wang X., Gao M., Guo B., Gao M., Liu J., Yu Y., Wang L., Kong W. (2021). Prediction of drug efficacy from transcriptional profiles with deep learning. Nat. Biotechnol..

[67] Wan F., Kontogiorgos-Heintz D., de la Fuente-Nunez C. (2022). Deep generative models for peptide design. Digit. Discov..

[68] Müller A., Hiss J.A., Schneider G. (2018). Recurrent Neural Network Model for Constructive Peptide Design. J. Chem. Inf. Model..

[69] Bolatchiev A., Baturin V., Shchetinin E., Bolatchieva E. (2022). Novel Antimicrobial Peptides Designed Using a Recurrent Neural Network Reduce Mortality in Experimental Sepsis. Antibiotics.

[70] Das P., Sercu T., Wadhawan K., Padhi I., Gehrmann S., Cipcigan F., Chenthamarakshan V., Strobelt H., dos Santos C., Chen P.-Y. (2021). Accelerated antimicrobial discovery via deep generative models and molecular dynamics simulations. Nat. Biomed. Eng..

[71] Schissel C.K., Mohapatra S., Wolfe J.M., Fadzen C.M., Bellovoda K., Wu C.-L., Wood J.A., Malmberg A.B., Loas A., Gómez-Bombarelli R. (2021). Deep learning to design nuclear-targeting abiotic miniproteins. Nat. Chem..

[72] Askr H., Elgeldawi E., Ella H.A., Elshaier Y.A.M.M., Gomaa M.M., Hassanien A.E. (2022). Deep learning in drug discovery: An integrative review and future challenges. Artif. Intell. Rev..

[73] Abbasi K., Razzaghi P., Poso A., Ghanbari-Ara S., Masoudi-Nejad A. (2021). Deep Learning in Drug Target Interaction Prediction: Current and Future Perspectives. Curr. Med. Chem..

[74] Zeng X., Zhu S., Lu W., Liu Z., Huang J., Zhou Y., Fang J., Huang Y., Guo H., Li L. (2020). Target identification among known drugs by deep learning from heterogeneous networks. Chem. Sci..

[75] Zhao Y., Zheng K., Guan B., Guo M., Song L., Gao J., Qu H., Wang Y., Shi D., Zhang Y. (2020). DLDTI: A learning-based framework for drug-target interaction identification using neural networks and network representation. J. Transl. Med..

[76] Irwin J.J., Sterling T., Mysinger M.M., Bolstad E.S., Coleman R.G. (2012). ZINC: A Free Tool to Discover Chemistry for Biology. J. Chem. Inf. Model..

[77] Sterling T., Irwin J.J. (2015). ZINC 15—Ligand Discovery for Everyone. J. Chem. Inf. Model..

[78] EBI Web Team ChEMBL. https://www.ebi.ac.uk/chembl/.

[79] Clarivate Analytics Integrity Integrity DataBase. https://integrity.clarivate.com/.

[80] ChemBridge The Gold Standard in Small Molecule Libraries and Building Blocks. https://chembridge.com/.

[81] Asinex. https://www.asinex.com/.

[82] Zhang Group GLASS: GPCR-Ligand Association Database. https://zhanggroup.org/GLASS/.

[83] Subramanian A., Narayan R., Corsello S.M., Peck D.D., Natoli T.E., Lu X., Gould J., Davis J.F., Tubelli A.A., Asiedu J.K. (2017). A next generation connectivity map: L1000 platform and the first 1,000,000 profiles. Cell.

[84] Rohrer S.G., Baumann K. (2009). Maximum Unbiased Validation (MUV) Data Sets for Virtual Screening Based on PubChem Bioactivity Data. J. Chem. Inf. Model..

[85] University of Nebraska Medical Center APD3 Antimicrobial Peptide Database. https://aps.unmc.edu/.

[86] UniPro. http://www.uniprot.org.

[87] Bhadra P., Yan J., Li J., Fong S., Siu S.W.I. (2018). AmPEP: Sequence-based prediction of antimicrobial peptides using distribution patterns of amino acid properties and random forest. Sci. Rep..

[88] Pirtskhalava M., Gabrielian A., Cruz P., Griggs H.L., Squires R.B., Hurt D.E., Grigolava M., Chubinidze M., Gogoladze G., Vishnepolsky B. (2015). DBAASP v.2: An enhanced database of structure and antimicrobial/cytotoxic activity of natural and synthetic peptides. Nucleic Acids Res..

[89] Gupta S., Kapoor P., Chaudhary K., Gautam A., Kumar R., Raghava G.P.S., Open Source Drug Discovery Consortium (2013). In Silico Approach for Predicting Toxicity of Peptides and Proteins. PLoS ONE.

[90] Agrawal P., Bhalla S., Usmani S.S., Singh S., Chaudhary K., Raghava G.P.S., Gautam A. (2015). CPPsite 2.0: A repository of experimentally validated cell-penetrating peptides. Nucleic Acids Res..

[91] Wishart D.S., Feunang Y.D., Guo A.C., Lo E.J., Marcu A., Grant J.R., Sajed T., Johnson D., Li C., Sayeeda Z. (2018). DrugBank 5. 0: A Major Update to the DrugBank Database for Nucleic Acids Res..

[92] Yang H., Qin C., Li Y.H., Tao L., Zhou J., Yu C.Y., Xu F., Chen Z., Zhu F., Chen Y.Z. (2015). Therapeutic target database update 2016: Enriched resource for bench to clinical drug target and targeted pathway information. Nucleic Acids Res..

[93] Hernandez-Boussard T., Whirl-Carrillo M., Hebert J.M., Gong L., Owen R., Gong M., Gor W., Liu F., Truong C., Whaley R. (2008). The pharmacogenetics and pharmacogenomics knowledge base: Accentuating the knowledge. Nucleic Acids Res..

[94] Cheng F., Li W., Wang X., Zhou Y., Wu Z., Shen J., Tang Y. (2013). Adverse Drug Events: Database Construction and in Silico Prediction. J. Chem. Inf. Model..

[95] Ctd Illuminating How Chemicals Affect Human Health. http://ctdbase.org/.

[96] SIDER 4.1. Side Effect Resource. http://sideeffects.embl.de/.

[97] Kuhn M., Campillos M., Letunic I., Jensen L.J., Bork P. (2010). A side effect resource to capture phenotypic effects of drugs. Mol. Syst. Biol..

[98] Arús-Pous J., Johansson S.V., Prykhodko O., Bjerrum E.J., Tyrchan C., Reymond J.-L., Chen H., Engkvist O. (2019). Randomized SMILES strings improve the quality of molecular generative models. J. Chemin-.

[99] Schroedl S. (2019). Current methods and challenges for deep learning in drug discovery. Drug Discov. Today Technol..

[100] Nag S., Baidya A.T.K., Mandal A., Mathew A.T., Das B., Devi B., Kumar R. (2022). Deep learning tools for advancing drug discovery and development. 3 Biotech.

[101] Pushpakom S., Iorio F., Eyers P.A., Escott K.J., Hopper S., Wells A., Doig A., Guilliams T., Latimer J., McNamee C. (2019). Drug repurposing: Progress, challenges and recommendations. Nat. Rev. Drug Discov..

[102] Song T., Wang G., Ding M., Rodriguez-Paton A., Wang X., Wang S. (2021). Network-Based Approaches for Drug Repositioning. Mol. Informatics.

[103] Staszak M., Staszak K., Wieszczycka K., Bajek A., Roszkowski K., Tylkowski B. (2021). Machine learning in drug design: Use of artificial intelligence to explore the chemical structure–biological activity relationship. WIREs Comput. Mol. Sci..

[104] Wang M., Wang Z., Sun H., Wang J., Shen C., Weng G., Chai X., Li H., Cao D., Hou T. (2022). Deep learning approaches for de novo drug design: An overview. Curr. Opin. Struct. Biol..

