# Supplementary material for: A Systematic Review of Deep Learning Methodologies Used in the Drug Discovery Process with Emphasis on In Vivo Validation
Source: Int J Mol Sci. 2023 Mar 31;24(7):6573. doi: 10.3390/ijms24076573 (PMC10095548; doi:10.3390/ijms24076573)
Supplement: Supplementary file 1 [file ijms-24-06573-s001.zip › ijms-2136933-supplementary.pdf]

Supplementary Material

List of selected papers after abstract screening:

[1] A. Bolatchiev, V. Baturin, E. Shchetinin, and E. Bolatchieva, “Novel Antimicrobial Peptides Designed Using a Recurrent Neural Network Reduce Mortality in Experimental Sepsis,” *Antibiotics*, vol. 11, no. 3, 2022, doi: 10.3390/antibiotics11030411.

[2] K. Bouhedjar, A. Boukelia, A. Khorief Nacereddine, A. Boucheham, A. Belaidi, and A. Djerourou, “A natural language processing approach based on embedding deep learning from heterogeneous compounds for quantitative structure-activity relationship modeling,” *Chem. Biol. Drug Des.*, vol. 96, no. 3, pp. 961–972, 2020, doi: 10.1111/cbdd.13742.

[3] D. V. Bozhko *et al.*, “Artificial intelligence-driven phenotyping of zebrafish psychoactive drug responses,” *Prog. Neuropsychopharmacol. Biol. Psychiatry*, vol. 112, 2022, doi: 10.1016/j.pnpbp.2021.110405.

[4] B. Chiovini *et al.*, “Theoretical design, synthesis, and in vitro neurobiological applications of a highly efficient two-photon caged GABA validated on an epileptic case,” *ACS Omega*, vol. 6, no. 23, pp. 15029–15045, 2021, doi: 10.1021/acsomega.1c01164.

[5] G. Choi, D. Kim, and J. Oh, “AI-Based Drug Discovery of TKIs Targeting L858R/T790M/C797S-Mutant EGFR in Non-small Cell Lung Cancer,” *Front. Pharmacol.*, vol. 12, 2021, doi: 10.3389/fphar.2021.660313.

[6] P. Das *et al.*, “Accelerated antimicrobial discovery via deep generative models and molecular dynamics simulations,” *Nat. Biomed. Eng.*, vol. 5, no. 6, pp. 613–623, 2021, doi: 10.1038/s41551-021-00689-x.

[7] D. R. Gawel *et al.*, “A validated single-cell-based strategy to identify diagnostic and therapeutic targets in complex diseases,” *Genome Med.*, vol. 11, no. 1, 2019, doi: 10.1186/s13073-019-0657-3.

[8] A. H. Göller, L. Kuhnke, A. Ter Laak, K. Meier, and A. Hillisch, “Machine Learning Applied to the Modeling of Pharmacological and ADMET Endpoints.,” *Methods Mol. Biol. Clifton NJ*, vol. 2390, pp. 61–101, 2022, doi: 10.1007/978-1-0716-1787-8_2.

[9] I. Icke *et al.*, “3D profiling of amyloid plaque-associated microglia and neuronal damage on confocal fluorescence images to aid drug discovery in Alzheimer’s disease,” in *Progress in Biomedical Optics and Imaging - Proceedings of SPIE*, 2019, vol. 10956. doi: 10.1117/12.2512452.

[10] H. Iwata *et al.*, “Prediction of Total Drug Clearance in Humans Using Animal Data: Proposal of a Multimodal Learning Method Based on Deep Learning,” *J. Pharm. Sci.*, vol. 110, no. 4, pp. 1834–1841, 2021, doi: 10.1016/j.xphs.2021.01.020.

[11] P. Jiang, S. Huang, Z. Fu, Z. Sun, T. M. Lakowski, and P. Hu, “Deep graph embedding for prioritizing synergistic anticancer drug combinations.,” *Comput. Struct. Biotechnol. J.*, vol. 18, pp. 427–438, 2020, doi: 10.1016/j.csbj.2020.02.006.

[12] V. Khanna, L. Li, J. Fung, S. Ranganathan, and N. Petrovsky, “Prediction of novel mouse TLR9 agonists using a random forest approach,” *BMC Mol. Cell Biol.*, vol. 20, 2019, doi: 10.1186/s12860-019-0241-0.

[13] S. Krishnan G, A. Joshi, N. Akhtar, and V. Kaushik, “Immunoinformatics designed T cell multi epitope dengue peptide vaccine derived from non structural proteome,” *Microb. Pathog.*, vol. 150, 2021, doi: 10.1016/j.micpath.2020.104728.

[14] I. Lee, J. Keum, and H. Nam, “DeepConv - DTI: prediction of drug-target interactions via deep learning with convolution on protein sequences,” *ArXivorg E-Print Arch. Quant. Biol.*, pp. 1–26, 2018.

[15] X. Li, Y. Xu, H. Yao, and K. Lin, “Chemical space exploration based on recurrent neural networks: Applications in discovering kinase inhibitors,” *J. Cheminformatics*, vol. 12, no. 1, 2020, doi: 10.1186/s13321-020-00446-3.

[16] X. Li, Z. Zhang, D. Xu, C. Wu, J. Li, and Y. Zheng, “A prediction method for animal-derived drug resistance trend using a grey-bp neural network combination model,” *Antibiotics*, vol. 10, no. 6, 2021, doi: 10.3390/antibiotics10060692.

[17] Z. Liu *et al.*, "Deep learning enables discovery of highly potent anti-osteoporosis natural products," *Eur. J. Med. Chem.*, vol. 210, 2021, doi: 10.1016/j.ejmech.2020.112982.
[18] A. Lopez-Cortes *et al.*, "In silico analyses of immune system protein interactome network, single-cell RNA sequencing of human tissues, and artificial neural networks reveal potential therapeutic targets for drug repurposing against COVID-19," *ChemRxiv*, pp. 1–25, 2020.
[19] H. Mamada, Y. Nomura, and Y. Uesawa, "Prediction Model of Clearance by a Novel Quantitative Structure-Activity Relationship Approach, Combination DeepSnap-Deep Learning and Conventional Machine Learning," *ACS Omega*, vol. 6, no. 36, pp. 23570–23577, 2021, doi: 10.1021/acsomega.1c03689.
[20] B. J. Neves *et al.*, "Deep Learning-driven research for drug discovery: Tackling malaria," *PLoS Comput. Biol.*, vol. 16, no. 2, 2020, doi: 10.1371/journal.pcbi.1007025.
[21] O. Obrezanova *et al.*, "Prediction of In Vivo Pharmacokinetic Parameters and Time-Exposure Curves in Rats Using Machine Learning from the Chemical Structure.," *Mol. Pharm.*, Apr. 2022, doi: 10.1021/acs.molpharmaceut.2c00027.
[22] C. K. Schissel *et al.*, "Deep learning to design nuclear-targeting abiotic miniproteins," *Nat. Chem.*, vol. 13, no. 10, pp. 992–1000, 2021, doi: 10.1038/s41557-021-00766-3.
[23] S. Schneckener *et al.*, "Prediction of Oral Bioavailability in Rats: Transferring Insights from in Vitro Correlations to (Deep) Machine Learning Models Using in Silico Model Outputs and Chemical Structure Parameters," *J. Chem. Inf. Model.*, vol. 59, no. 11, pp. 4893–4905, 2019, doi: 10.1021/acs.jcim.9b00460.
[24] V. Singh, S. Shrivastava, S. Kumar Singh, A. Kumar, and S. Saxena, "Accelerating the discovery of antifungal peptides using deep temporal convolutional networks.," *Brief. Bioinform.*, vol. 23, no. 2, Mar. 2022, doi: 10.1093/bib/bbac008.
[25] N. Sobhani, "Novel, smaller and more powerful CTLA4 inhibitors for the treatment of solid tumors," in *Abstracts of Papers, 261st ACS National Meeting, April 5-16, 2021*, 2021.
[26] J. M. Stokes *et al.*, "A Deep Learning Approach to Antibiotic Discovery," *Cell Camb. MA U. S.*, vol. 180, no. 4, pp. 688-702.e13, 2020, doi: 10.1016/j.cell.2020.01.021.
[27] X. Tan *et al.*, "Automated design and optimization of multitarget schizophrenia drug candidates by deep learning," *Eur. J. Med. Chem.*, vol. 204, p. 112572, 2020, doi: 10.1016/j.ejmech.2020.112572.
[28] X. Tan *et al.*, "Discovery of Pyrazolo[3,4-d]pyridazinone Derivatives as Selective DDR1 Inhibitors via Deep Learning Based Design, Synthesis, and Biological Evaluation.," *J. Med. Chem.*, vol. 65, no. 1, pp. 103–119, Jan. 2022, doi: 10.1021/acs.jmedchem.1c01205.
[29] H. Wang, M. Xie, G. Rizzi, X. Li, K. Tan, and M. Fussenegger, "Identification of Sclareol As a Natural Neuroprotective Cav 1.3-Antagonist Using Synthetic Parkinson-Mimetic Gene Circuits and Computer-Aided Drug Discovery.," *Adv. Sci. Weinh. Baden-Wurtt. Ger.*, p. e2102855, 2022, doi: 10.1002/advs.202102855.
[30] J. Williams *et al.*, "Using in vitro ADME data for lead compound selection: An emphasis on PAMPA pH 5 permeability and oral bioavailability," *Bioorg. Med. Chem.*, vol. 56, p. 116588, 2022, doi: 10.1016/j.bmc.2021.116588.
[31] Y. Yang *et al.*, "Discovery of Highly Potent, Selective, and Orally Efficacious p300/CBP Histone Acetyltransferases Inhibitors," *J. Med. Chem.*, vol. 63, no. 3, pp. 1337–1360, 2020, doi: 10.1021/acs.jmedchem.9b01721.
[32] X. Zeng *et al.*, "Target identification among known drugs by deep learning from heterogeneous networks.," *Chem. Sci.*, vol. 11, no. 7, pp. 1775–1797, Jan. 2020, doi: 10.1039/c9sc04336e.
[33] H. Zhang *et al.*, "A novel virtual screening procedure identifies Pralatrexate as inhibitor of SARS-CoV-2 RdRp and it reduces viral replication in vitro," *PLoS Comput. Biol.*, vol. 16, no. 12 December, 2020, doi: 10.1371/journal.pcbi.1008489.
[34] Y. Zhao *et al.*, "DLDTI: a learning-based framework for drug-target interaction identification using neural networks and network representation," *J. Transl. Med.*, vol. 18, no. 1, 2020, doi: 10.1186/s12967-020-02602-7.

[35] A. Zhavoronkov *et al.*, "Deep learning enables rapid identification of potent DDR1 kinase inhibitors," *Nat. Biotechnol.*, vol. 37, no. 9, pp. 1038–1040, 2019, doi: 10.1038/s41587-019-0224-x.
[36] J. Zhu *et al.*, "Prediction of drug efficacy from transcriptional profiles with deep learning," *Nat. Biotechnol.*, vol. 39, no. 11, pp. 1444–1452, 2021, doi: 10.1038/s41587-021-00946-z.
